# An mRNA-based FimH nanoparticle vaccine against uropathogenic *Escherichia coli* is highly immunogenic in rodents

**DOI:** 10.3389/fimmu.2025.1668937

**Published:** 2025-11-03

**Authors:** Sandro Roier, Roberto Adamo, Roberto Rosini, Alfredo Pezzicoli, Maria Scarselli, Benjamin Petsch, Edith Jasny, Susanne Rauch

**Affiliations:** ^1^ CureVac SE, Tübingen, Germany; ^2^ GSK, Siena, Italy

**Keywords:** mRNA vaccine, uropathogenic *E. coli*, UPEC, UPEC vaccine, FimH, nanoparticle, urinary tract infection

## Abstract

**Background:**

Uropathogenic *Escherichia coli* (UPEC) is the leading cause of urinary tract infections (UTIs), which are increasingly antibiotic resistant and frequently recur. Novel therapeutics are sought to treat and prevent recurrent UTIs (rUTIs), including vaccines. Key virulence factor FimH, which mediates bacterial adhesion to host cells and biofilm formation, is a promising target for a vaccine against UPEC. We assessed the immunogenicity of mRNA-based nanoparticle vaccines against UPEC containing FimH as the encoded antigen.

**Methods:**

Lipid nanoparticle (LNP)-formulated mRNA vaccines encoding FimH as a monomeric, pre-binding conformation protein (FimH_DG_), or a multimeric protein nanoparticle (PNP) through fusion to *Helicobacter pylori* ferritin (FimH_DG_-Ferritin) were developed. Immunogenicity was assessed *in vivo* in female BALB/cAnNRj mice and female Wistar rats following three intramuscular (IM) injections of FimH_DG_ or FimH_DG_–Ferritin mRNA vaccines, or comparator protein subunit vaccines. Antibody levels and functional response were measured in serum and urine by ELISA and bacterial adhesion inhibition (BAI) assays. T cell response was characterized by flow cytometry.

**Results:**

In both animal models, unmodified FimH_DG_ and FimH_DG_-Ferritin mRNA vaccines induced higher functional serum antibody levels compared with the protein subunit vaccine control, at the tested dosages. FimH_DG_-Ferritin resulted in greater binding antibody levels and higher splenic FimH-specific CD4^+^ and CD8^+^ T cell responses compared with monomeric FimH_DG_ in both models, resulting in its nomination as lead candidate vaccine design. Validation in rats demonstrated that N1mΨ nucleoside modification further enhanced FimH_DG_-Ferritin immunogenicity compared with unmodified mRNA.

**Conclusions:**

The mRNA vaccine FimH_DG_-Ferritin with N1mΨ-modified nucleosides is a promising candidate for further development as a vaccine against UPEC.

## 1 Introduction

Urinary tract infections (UTIs) are among the most common bacterial infections globally, with over 404 million cases reported in 2019 (1). In the US alone, the annual direct healthcare costs associated with UTIs are estimated to be around $2 billion (2). UTIs are significantly more common in women than in men; the lifetime incidence is 50−60% in adult women compared with just 12% in men (3, 4). Current medical approaches for the management of UTIs include prophylactic measures, over-the-counter medications, or treatment with antibiotics (5). Despite these measures, around one quarter of women will experience another UTI within six months of treatment for the initial infection (6). The occurrence of two or more UTIs within six months, or three or more UTIs within a year, is defined as recurrent UTI (rUTI) (5).

The most common causative agent of UTIs is uropathogenic *Escherichia coli* (UPEC), accounting for approximately 75% of cases (7). In general, the bacteria are transmitted to the urinary tract through fecal shedding and ascend the urinary tract to invade and colonize the bladder (cystitis) and kidneys (pyelonephritis) (8). The formation of biofilm-like intracellular bacterial communities in the host tissue contributes to the recurrence of UTIs within a short period of time (9, 10). Moreover, the prevalence of antimicrobial resistance is a major obstacle to successful treatment of UTIs caused by UPEC (11). Indeed, resistance to common antibiotics has been found in 29.7–46.2% of UPEC-derived rUTIs (12). In response, whole cell vaccines against UPEC have been developed, including Uro-Vaxom/OM-89, Solco-Urovac, and Uromune/MV140 (13–15). However, a recent systematic review found limited evidence of their long-term efficacy for reducing rUTIs in adult female patients (16) and while they are available in some countries, no vaccine to prevent rUTI infection has received FDA approval to date (17). Therefore, there is a significant unmet need for an efficacious vaccine targeting UPEC.

In this work, we investigated the immunogenicity of lipid nanoparticle (LNP)-formulated messenger ribonucleic acid (mRNA)-based candidate vaccines against UPEC. Developed using CureVac’s proprietary RNActive^®^ technology, these vaccine candidates encode the pilin FimH, a highly conserved key virulence factor of UPEC that is displayed at the tip of type 1 pili and mediates both biofilm formation and bacterial adhesion to host tissue (8, 18–20). The N-terminal lectin domain of FimH (FimH_L_) binds to mannosylated proteins (e.g. uroplakins) expressed on the surface of uroepithelial cells (8, 21). The C-terminal pilin domain of FimH possesses a hydrophobic groove that can impact protein conformation by interaction with different proteins, including the bacterial proteins FimG and FimC that hold the protein in a pre-and post-binding conformation, respectively (8, 21–23). In the pre-binding conformation, FimH transitions between low- and high-affinity binding modes through an equilibrium between tense and relaxed conformation states (8). By fusing a donor strand peptide of FimG to FimH (FimH_DG_), vaccine candidates investigated here enable the expression of FimH stabilized in an immunologically preferred pre-binding conformation (24). Vaccines were either designed to encode a secreted monomeric protein (FimH_DG_) or a secreted protein that self-assembles into a multimeric protein nanoparticle (PNP) by encoding *Helicobacter pylori* (*H. pylori*) ferritin fused to FimH_DG_ (FimH_DG_-Ferritin).

Ferritin PNPs have shown promise as delivery systems to increase the efficacy and safety of various therapeutic applications, such as anti-cancer drugs and novel prophylactic vaccines (25–29). Ferritin PNPs can present antigens on their surface, as seen with SARS-CoV-2 Spike and influenza stem hemagglutinin antigens (26, 28, 30–32). Protein-based approaches have shown broad immunogenicity and longer blood half-life of ferritin PNPs compared to antigens alone (30, 31, 33). *H. pylori* ferritin has also been employed as a fusion domain in mRNA vaccine development, improving antigenicity and immunogenicity of vaccines in animal models of SARS-CoV-2 (34) and HIV-1 (35). With good potency, safety and efficacy, mRNA vaccines are promising alternatives to traditional vaccines (36), highlighting the potential synergy that can be achieved in a vaccine platform harnessing both mRNA and ferritin PNP technology.

Existing strategies in UPEC vaccine development have largely involved protein subunit vaccines (37). These include the FimCH vaccine, where a complex of the chaperone FimC and FimH is formulated with a synthetic adjuvant, Phosphorylated HexaAcyl Disaccharide (PHAD™). In a Phase 1 study, the FimCH vaccine was able to reduce the frequency of UTIs in a subset of women with rUTI following administration of four doses (38). However, to our knowledge, no mRNA-based vaccines encoding antigens clustering on *H. pylori* ferritin PNPs have been clinically tested to date. Here, the mRNA vaccine candidates FimH_DG_ and FimH_DG_-Ferritin (including unmodified or N1-methyl-pseudouridine (N1mΨ)-modified nucleosides) were evaluated *in vivo* in mice and rats for induction of FimH-specific humoral and/or cellular immune responses, using PHAD-adjuvanted FimHC and AS01-adjuvanted FimH_DG_ protein subunit vaccines as controls (39).

## 2 Materials and methods

### 2.1 mRNA vaccines

mRNA vaccines were developed with CureVac’s RNActive^®^ platform and contained either chemically unmodified nucleosides or N1mΨ-modified nucleosides. Vaccines comprised of a 5′ cap1 structure, a 5′ untranslated region (UTR) from the human hydroxysteroid 17-beta dehydrogenase 4 gene (*HSD17B4*), a GC-enriched open reading frame (ORF), a 3′ UTR from the human proteasome 20 S subunit beta 3 gene (*PSMB3*), a histone stem-loop, and a poly(A) tail. LNP encapsulation of mRNA was performed using LNP technology from Acuitas Therapeutics (Canada). LNPs were composed of an ionizable amino lipid, phospholipid, cholesterol, and a PEGylated lipid. The FimH_DG_ mRNA vaccine encoded an N-terminal signal peptide from the mouse Igκ light chain (amino acid [aa] 1–20; NCBI reference sequence: AAH80787.1) followed by FimH from *E. coli* strain J96 (aa 22–300; NCBI reference sequence: ELL41155.1), a 5-aa linker (PGDGN), and the donor strand peptide of FimG from *E. coli* strain J96 (denoted as DG; aa 24–37; NCBI reference sequence: ELL41154.1). The FimH_DG_-Ferritin mRNA vaccines encoded FimH_DG_ with a different signal peptide (human IgE; aa 1–18; NCBI reference sequence: AAB59424.1) N-terminally linked via serine-glycine-glycine (SGG) to the ferritin domain of *H. pylori* strain J99 (aa 5-167; NCBI reference sequence: AAD06160.1) containing an N19Q mutation to remove a potential N-linked glycosylation site (33). The immunogenicity of FimH mRNA vaccines containing either Igκ- or IgE-derived signal peptides was tested in mice, and no significant differences were found based on the choice of signal peptide (39).

### 2.2 Protein vaccines

The PHAD-adjuvanted FimHC (also designated as FimCH) protein subunit vaccine consisted of a complex of FimH and FimC and was obtained as previously described (40). The AS01-adjuvanted FimH_DG_ (also designated as FimHdG) protein subunit vaccine employed an aa sequence that corresponds to the encoded sequence of the FimH_DG_ mRNA vaccine (without a signal peptide) and was obtained as previously described (39).

### 2.3 Animal ethics statement

Animal research in this study is reported in accordance with ARRIVE reporting guidelines (41). Female BALB/cAnNRj mice (Janvier Labs, France) and female Wistar rats (Charles River Laboratories, Germany) were provided and handled by Preclinics Gesellschaft für präklinische Forschung mbH (Germany). All in-life experimental procedures undertaken during the course of the mouse and rat immunization studies were conducted in accordance with German laws and guidelines for animal protection, appropriate local and national approvals, and with Directive 2010/63/EU of the European Parliament and of the Council of 22 September 2010 on the protection of animals used for scientific purposes. Ethical approval was obtained from Land Brandenburg, Landesamt für Arbeitsschutz, Vebraucherschutz und Gesundheit (Germany) for the mouse (reference number: 2347-14-2018) and rat (reference number: 2347-5-2021) studies. Animals were acclimated for at least one week before any procedures were carried out and were 6–7 weeks old at the start of the study. Mice were kept in Macrolon type II cages (4 mice per cage) and rats were kept in Macrolon type IV cages (3–4 rats per cage). Both were housed at a temperature of 19–23°C, humidity of 35–50%, 15 air changes per hour and a 12/12-hour light/dark cycle. Diets consisted of Ssniff R/M-H extruded (V1536); both food and water were provided *ad libitum.*

### 2.4 Vaccination studies

A total of 158 animals were used in these studies (32 mice [n=8/group]; 70 rats in the first study [n=7/group]; 56 rats in the second study [n=8/group]). The sample size was selected to be as low as possible in each study while still providing sufficient power to determine between-group differences, based on the authors’ experience and knowledge from previous mouse and rat studies. Animals were assigned to groups at random on arrival and the studies were not blinded.

mRNA vaccine dosages used were decided based on accumulated learnings from previous studies performed by CureVac or others (38–40), and the protein subunit vaccine dosage was used at a similar level to the mRNA dosages. Of note, equal doses of protein and mRNA vaccine injected are not expected to result in equivalent amounts of antigen presented *in vivo*, since mRNA-based expression is highly dependent on the cell type-specific quality of mRNA vaccine uptake, antigen expression and/or secretion (42, 43).

On Days 0, 21, and 35, female BALB/cAnNRj mice (n=8/group) received an intramuscular (IM) injection (50 µL; 25 µL each into the left and right *M. tibialis*) of either: 2 µg FimHC protein complex subunit vaccine adjuvanted with 4 µg PHAD; 2 µg of FimH_DG_ mRNA vaccine (unmodified nucleosides); or 2 µg of FimH_DG_-Ferritin mRNA vaccine (unmodified nucleosides) using a single-use insulin syringe with an integrated 30G needle (BD, Cat. 324825). Mice receiving physiological saline (25 µL of 0.9% NaCl into left or right *M. tibialis*) served as negative controls. The animals in each group were divided into two cohorts (n=4/cohort), and the experiment was started on two consecutive days. Blood samples were collected into Z-clot activator microtubes (Sarstedt, Cat. 20.1291) by retro-orbital bleeding on Days 21, 35, and 49 under inhalation anesthesia using isoflurane (5 vol%). The samples were incubated for 0.5–1 hour (h) at room temperature (RT), and sera were obtained by collecting the supernatant after centrifugation (5 minutes [min]; 10,000 × g; RT). Spontaneous urine was collected on Days 21 and 35 by placing the animals in a cage with hydrophobic Medicat LabSand™ bedding (WDT, Cat. 27906) and collecting urine directly with a pipette. On Day 49, urine was collected by puncture of the bladder to ensure the collection of a larger sample volume. Urine was centrifuged (5 min; 10,000 × g; RT) and the supernatant used for analysis. At the end of the study on Day 49, mice were euthanized by exsanguination under inhalation anesthesia using isoflurane (5 vol%).

Splenocytes were isolated by mechanical disruption of the spleen using 40 µm cell strainers (Pluriselect, Cat. 43-50040-51). Red blood cells were lysed and splenocytes were washed, resuspended in supplemented medium and finally stored as single-cell suspensions in liquid nitrogen until use.

For the first rat study, female Wistar rats (n=7/group) were injected IM three times (100 µL each into the left or right *M. gastrocnemius*) on Days 0, 21, and 35 with either: 0.7 µg, 2.8 µg or 8.5 µg FimH_DG_ protein subunit vaccine adjuvanted with 2.5 µg AS01 each; 1 µg, 4 µg or 12 µg FimH_DG_ mRNA vaccine; or 1 µg, 4 µg or 12 µg FimH_DG_-Ferritin mRNA vaccine containing unmodified nucleosides. In the second rat study, female Wistar rats (n=8/group) were injected IM three times (100 µL each into the left or right *M. gastrocnemius*) on Days 0, 21, and 35 with either: 1 µg or 12 µg FimH_DG_ protein subunit vaccine adjuvanted with 2.5 µg AS01 each, or 1 µg or 12 µg of FimH_DG_-Ferritin mRNA vaccines containing either unmodified or N1mΨ-modified nucleosides. Injections were performed using a single-use insulin syringe with an integrated 30G needle (BD, Cat. 324825) and rats receiving physiological saline (100 µL of 0.9% NaCl into left or right *M. gastrocnemius*) served as negative controls.

Blood samples were collected on Days 21 and 35 by retro-orbital bleeding into Multivette 600 Z-Gel tubes (Sarstedt, Cat. 15.1674) and on Day 49 by heart puncture into Vacuette tubes (8 mL CAT Serum Sep Clot Activator; Greiner Bio One, Cat. 455071) under inhalation anesthesia using isoflurane (5 vol%). The samples were incubated for 0.5–1 h at RT, and sera were then obtained by collecting the supernatant after centrifugation (5 min; 10,000 × g; RT). Spontaneous urine was collected on Days 21 and 35 by placing the animals in an empty cage and urine collected directly with a pipette if the animals urinated. If no urine could be gained, the abdomen was palpated whilst under anesthesia for test substance administration. In case of a palpable bladder, the urine was gained by gentle pressure on the bladder. On Day 49, urine was collected by puncture of the bladder. Urine was centrifuged (5 min; 10,000 × g; RT) and the supernatant used for analysis. At the end of the study on Day 49, rats were euthanized by exsanguination under inhalation anesthesia using isoflurane (5 vol%).

Mice and rats were visually inspected at least once daily for evidence of ill-health. The injection sites were monitored daily after each injection for swelling, reddening or other adverse effects until no deviations from the normal condition could be detected. Body weights were obtained on Days 0, 1, 2, 7, 14, 21, 22, 28, 35, 36, 42 and 49. Body weight loss of more than 20% was established *a priori* as criteria for exclusion from study; no animals were excluded.

### 2.5 Quantification of binding antibodies

Quantities of FimH_L_-specific binding antibodies (total IgG) in sera and urine samples of vaccinated mice or rats were assessed by indirect enzyme-linked immunosorbent assay (ELISA). 96-well MaxiSorp ELISA plates (black; ThermoFisher Scientific, Cat. 437111) were coated with 1 µg/mL of a recombinant FimH_L_ protein overnight at 4 °C. FimH_L_ was obtained as previously described (39). Plates were washed and blocked for 2 h at 37 °C with 5% milk in phosphate-buffered saline (PBS)/0.05% Tween-20 for mouse samples and 1% bovine serum albumin (BSA) in PBS/0.05% Tween-20 for rat samples. Serum samples were added in serial dilution and incubated for 2 h at RT. For mouse urine, the FimH_L_-specific binding antibody levels were determined in two pools of four animals each due to the small amount of urine; whereas, rat urine samples were tested per animal. For sample preparation, the pH value of each mouse urine pool or rat urine sample was measured. If the pH value was <7, urine samples were neutralized by mixing the samples 1:1 with PBS (pH 8.2). If the pH value was ≥7, urine samples were directly diluted 1:2 in blocking buffer. Dilution in PBS (pH 8.2) or blocking buffer was performed directly as a predilution before urine samples were added in serial dilution and incubated for 2 h at RT. After washing, plates were incubated with either horseradish peroxidase (HRP)-conjugated goat anti-mouse IgG (H+L) (1:5000; Jackson-Immuno Research, Cat. 115-035-003) or HRP-conjugated goat anti-rat IgG (1:5000; Sigma Aldrich, Cat. A9037) in blocking buffer for 1–1.5 h at RT. Finally, plates were washed, Amplex™ UltraRed reagent (1:200; Invitrogen, Cat. A36006) with 30% H_2_O_2_ (1:2000; Fluka, Cat. 95302) was added, and fluorescence was detected after 45–90 min using a BioTek SynergyHTX plate reader (excitation 530/25, emission detection 590/35, sensitivity 45; Agilent BioTek). The ELISA endpoint titers were defined as the highest reciprocal serum or urine dilution that yielded a signal above the mean background signal plus 5-fold standard deviation (SD).

### 2.6 T cell analysis

The induction of antigen-specific T cells was determined using intracellular cytokine staining in combination with flow cytometry. Mouse splenocytes were thawed and 2 x 10^6^ cells per well (200 µL) were stimulated for 6–7 h at 37 °C with a custom-made 15-mer overlapping (11 aa) peptide library (1 µg/mL for each peptide) covering the full-length FimH in the presence of anti-CD28 (1:400; BD Biosciences, Cat. 553294) in α-MEM medium (Gibco, Cat. 22561) with 10% FCS (HyClone, Cat. 30160.03), 100 U/mL penicillin/100 mg/mL streptomycin (Lonza, Cat. DE17-602E), 2 mM L-glutamine (Lonza, Cat. BE17-605E) and 10mM HEPES (CureVac SE). After 1 h, GolgiPlug™ (BD Biosciences, Cat. 555029) was added in a dilution of 1:200 (50 µL) to the splenocytes to inhibit cytokine secretion. After stimulation, splenocytes were centrifuged, resuspended in supplemented α-MEM medium (Gibco, Cat. 22561), and stored overnight at 4 °C. On the following day, splenocytes were washed twice in PBS and stained with LIVE/DEAD™ fixable aqua dead cell stain kit (Invitrogen, Cat. L34957) for 30 min at 4 °C in the dark. After an additional washing step in PBS with 0.5% BSA, cells were surface stained for Thy1.2 (FITC rat anti-mouse CD90.2 [Thy1.2]; 1:200; BioLegend, Cat. 140304), CD4 (V450 rat anti-mouse CD4; 1:200; BD Biosciences, Cat. 560468) and CD8 (APC-Cy7 rat anti-mouse CD8a; 1:200; BioLegend, Cat. 100714) and incubated with FcγR-block (rat anti-mouse CD16/CD32; 1:100; Invitrogen, Cat. 14-0161-85) in PBS with 0.5% BSA for 30 min at 4 °C in the dark. Splenocytes were then washed and fixed using Cytofix/Cytoperm™ solution (BD Biosciences, Cat. 554722) for 20 min at RT in the dark. After fixation, cells were washed in permeabilization buffer (PBS, 0.5% BSA, 0.1% Saponin) and stained for interferon (IFN)-γ (APC rat anti-mouse IFN-γ; 1:100; BD Biosciences, Cat. 554413) and tumor necrosis factor (TNF; Phycoerythrin [PE]-conjugated, rat anti-mouse TNF alpha, 1:100; Invitrogen, Cat. 12-7321-82) for 30 min at 4 °C in the dark. Splenocytes were subsequently washed in permeabilization buffer and resuspended in PBEA buffer (PBS, 0.5% BSA, 2 mM EDTA, 0.01% sodium azide). Finally, splenocytes were analyzed by flow cytometry on a ZE5 flow cytometer (Bio-Rad Laboratories, Inc.) and data were analyzed using FlowJo™ software version 10.7.2 (Tree star, Inc.; Ashland, OR, USA).

### 2.7 Bacterial adhesion inhibition assay

Functional antibody responses were assessed by bacterial adhesion inhibition (BAI) assay as previously described (39). Briefly, a UPEC strain [UTI89; (44)] engineered to express the mCherry fluorescent marker was incubated for 30 min with monolayers of human uroepithelium cell line SV-HUC-1 (ATCC) in 96-well plates (ATCC, Cat. CRL-9520) in the presence of serially diluted sera from vaccinated or sham-vaccinated mice or rats. As positive and negative controls, 20% D-(+)-mannose and medium were used, respectively. After adhesion, cells were washed extensively to remove unbound bacteria and fixed with formaldehyde. Finally, the specific fluorescent signal associated with the adhered bacteria was recorded using an automated high content screening microscope (Opera Phenix) and quantified with Harmony software (Opera Phenix, version 5.1). The BAI titers were determined as the reciprocal serum dilution leading to 50% bacterial adhesion inhibition, indicated by the inflection point of the dose-response curve.

### 2.8 *In vitro* protein expression

**Figure 1:**
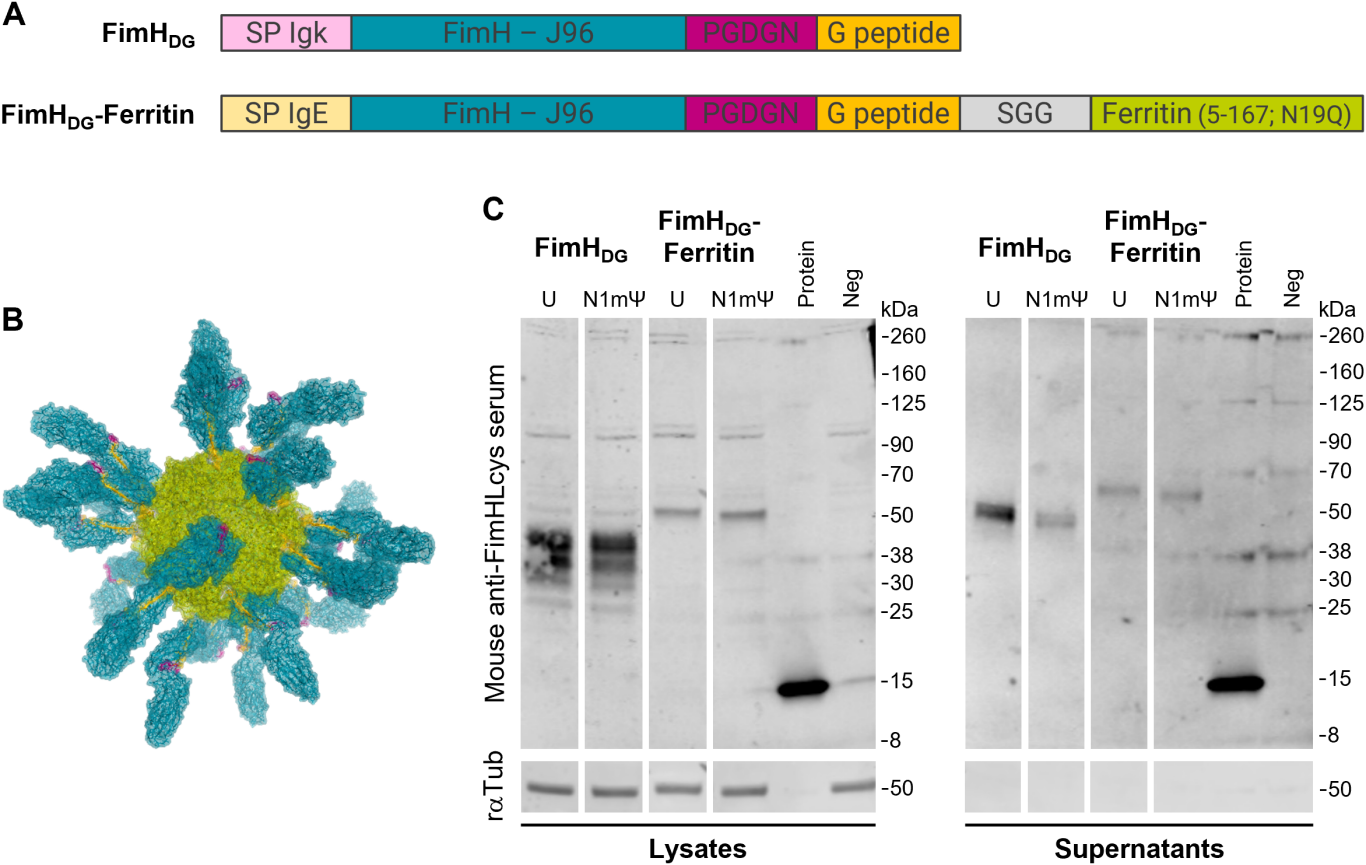
Proteins translated from mRNAs encoding FimH_DG_ or FimH_DG_-Ferritin are secreted in cell culture. **(A)** Schematic representation (depiction not to scale) of the open reading frames (ORFs) of mRNAs encoding FimH_DG_ or FimH_DG_-Ferritin. The ORFs include a signal peptide (SP IgE or SP Igk) at the N-terminus, followed by FimH from *E*. *coli* strain J96 (FimH - J96), a 5-amino acid (aa) linker (PGDGN), and the donor strand peptide of FimG (G peptide). The ORF of FimH_DG_-Ferritin contains an additional 3-aa linker (SGG) and the ferritin domain from *H*. *pylori* strain J99 (Ferritin [5-167; N19Q]) at the C-terminus. **(B)** Predicted structure of an mRNA-derived FimH_DG_-Ferritin protein nanoparticle (PNP). The expressed fusion protein FimH_DG_-Ferritin is expected to be presented to cells of the immune system as secreted self-assembled 24-mer PNPs. Each PNP is expected to consist of a ferritin core (green) displaying 24 copies of FimH (blue) stabilized in a pre-binding conformation by the PGDGN-linked (pink) donor strand peptide of FimG (yellow) on its surface. FimH_DG_-Ferritin denotes the fusion of FimH with the donor strand peptide of FimG (DG) and the *H*. *pylori* ferritin domain using an SGG-linker (grey). **(C)** HEK 293T cells were transfected with LNP-formulated mRNA containing either unmodified (U) or modified (N1mΨ) nucleosides encoding either FimH_DG_ or FimH_DG_-Ferritin. FimH expression in cell lysates and supernatants was analyzed via western blotting 48 h post transfection. HEK 293T cells incubated with medium served as negative control (Neg) and 100 ng recombinant FimH_L_ protein loaded to the gels was used as positive control (Protein). Rabbit anti-tubulin antibody (rαTub) was employed as loading control. Uncropped images are shown in **Supplementary Figure S1**.

For expression analysis of LNP-formulated mRNA encoding either FimH_DG_ or FimH_DG_-Ferritin containing either unmodified or modified (N1mΨ) nucleosides, HEK 293T cells were seeded at a density of 4 x 10^5^ cells/well in 6-well-plates (Sarstedt, Cat. 83.3920.300). The next day, cells were transfected under serum-free conditions with 0.5 µg of the respective mRNA vaccine per well. Protein expression in cell culture supernatants or cell lysates was assessed 48 h post transfection via SDS-PAGE (45) and western blotting. Supernatants were harvested and residual cells removed by centrifugation (500 × g, 3 min). Cells were lysed using RIPA with NP-40. Laemmli sample buffer (6x) was added to the supernatants and lysates before heating for 5 min at 95 °C. Equal sample volume (12.5 µl) was loaded per lane and proteins were separated on 4–20% Mini-PROTEAN^®^ TGX™ Precast Protein Gels (Bio-Rad, Cat.456-1095) before transfer to a nitrocellulose membrane (Odyssey^®^ nitrocellulose membrane, pore-size: 0.22 µm; LI-COR, Cat. 926-31092). Specific proteins were detected using mouse anti-FimHLcys polyclonal antiserum (1:1,000; obtained as previously described (39) and rabbit anti-alpha/beta tubulin antibody (1:1,000; Cell Signaling, Cat. 2148S), followed by goat anti-mouse IgG IRDye^®^ 800CW (1:10,000; LI-COR, Cat. 926-32210) and goat anti-rabbit IgG IRDye^®^ 680RD (1:10,000; LI-COR, Cat. 926-68071), respectively. Protein detection and image processing were carried out in an Odyssey^®^ CLx Imaging System and LI-COR’s Image Studio™ Lite version 5.2.5 according to the manufacturer’s instructions. All blots displayed in **Figure 1C** derive from the same experiment and were processed in parallel.

### 2.9 Human peripheral blood mononuclear cell stimulation and measurement of human IFN-α

Human peripheral blood mononuclear cells (hPBMCs) were isolated from buffy coats obtained from healthy adult donors through the blood bank in Tübingen (ZKT Tübingen gGMbH). Donors gave informed written consent for the use of their blood products in research and were anonymized prior to transfer to CureVac. Buffy coats were processed within 24 h of collection. Each buffy coat was diluted 1:1 with PBS and layered over Ficoll-Paque PLUS (Cytiva former GE Healthcare, Cat. 17-1440-02) for density gradient centrifugation at 805 × g for 20 min at RT without brake. The hPBMC layer was collected, washed twice with PBS, and cells were counted using a hemocytometer and trypan blue. Isolated hPBMCs were cryopreserved in FCS (HyClone, Cat. 30160.03) containing 10% DMSO (Sigma-Aldrich, Cat. 41639) and stored in liquid nitrogen. For stimulation, cryopreserved hPBMCs were thawed and transferred into 37 °C pre-warmed cell medium RPMI 1640 (Gibco, Cat. 52400025) with 20% FCS, 100 U/mL penicillin/100 mg/mL streptomycin (Lonza, Cat. DE17-602E), 2 mM L-glutamine (Lonza, Cat. BE17-605E). The cells were washed once, reconstituted, and 4 x 10^5^ cells were seeded per well into 96-well flat-bottom cell culture plate (Sarstedt, Cat. 83.3924). hPBMCs from three donors were incubated each in triplicates with 10 µg/mL of FimH_DG_-Ferritin mRNA vaccines containing either unmodified or modified (N1mΨ) nucleosides, prediluted in medium without FCS, in a total volume of 200 µL. hPBMCs treated with medium were used as control. After 24 h, cell-free supernatants were collected and analyzed in a 1:20 or 1:40 dilution using an IFN-α ELISA kit (PBL, Cat. 41115-1) according to the manufacturer’s instructions.

### 2.10 Statistical analyses

For the mouse and rat studies, differences between vaccines were assessed for each relevant readout using Analysis of Variance (ANOVA); different days were analyzed separately. For the rat study, the analysis accounted for both vaccine type and dose level, as well as the interaction between these two factors (vaccine*dose) to account for different potency between vaccines across the doses tested. If a significant difference between the vaccines was identified (assuming a significance level of α = 0.05), a *post-hoc* pairwise comparison test was performed to identify the differences using the Least Significant Difference (LSD) method. The corresponding *P* values are represented in the respective graphs.

All binding antibody data were log_10_-transformed for analysis. Normality was tested using the Shapiro-Wilk test, and subsequently by examination of QQ plots. Due to low granularity of values, in some cases Shapiro-Wilk indicated possibly non-normal data, but the corresponding QQ plots demonstrated little to no bias, suggesting underlying normality. In such cases, the Scheirer-Ray-Hare test was used to confirm the ANOVA results. Sphericity was tested using Mauchly’s test and was met in all cases. All statistical analyses were conducted in R (version 4.4.0 or higher, R Foundation for Statistical Computing) with Rstudio as the graphic user interface. The ANOVA was carried out using the aov function from the stats package and *post-hoc* tests using the PostHocTest function from the DescTools package.

## 3 Results

### 3.1 Proteins translated from mRNAs encoding FimH_DG_ or FimH_DG_-Ferritin are secreted in cell culture

The ORFs of the mRNAs encoding FimH_DG_ or FimH_DG_-Ferritin are depicted in **Figure 1A**. Both ORFs include a signal peptide (SP; SP Igk or SP IgE, respectively) at the N-terminus, followed by FimH from *E. coli* strain J96 (FimH – J96), a 5-aa linker (PGDGN), and the donor strand peptide of FimG (G peptide). The ORF of FimH_DG_-Ferritin contains an additional 3-aa linker (SGG) and the ferritin domain from *H. pylori* strain J99 (Ferritin [5-167; N19Q]) at the C-terminus. Antigen display on the ferritin surface enables uniform presentation of 24 copies of the antigen (46). The expressed fusion protein FimH_DG_-Ferritin is therefore expected to be presented to cells of the immune system as secreted self-assembled 24-mer PNPs, with FimH stabilized in a pre-binding conformation by the donor strand peptide of FimG on its surface (**Figure 1B**) (24).

Western blot analysis of cells transfected with LNP-formulated mRNA containing either unmodified (U) or modified (N1mΨ) nucleosides encoding either FimH_DG_ or FimH_DG_-Ferritin confirmed that mRNA encoding FimH_DG_ or FimH_DG_-Ferritin supported efficient expression and secretion of FimH (**Figure 1C**; **Supplementary Figure S1**).

### 3.2 FimH_DG_ and FimH_DG_-Ferritin mRNA vaccine candidates induce robust humoral and cellular immune responses in mice

**Figure 2:**
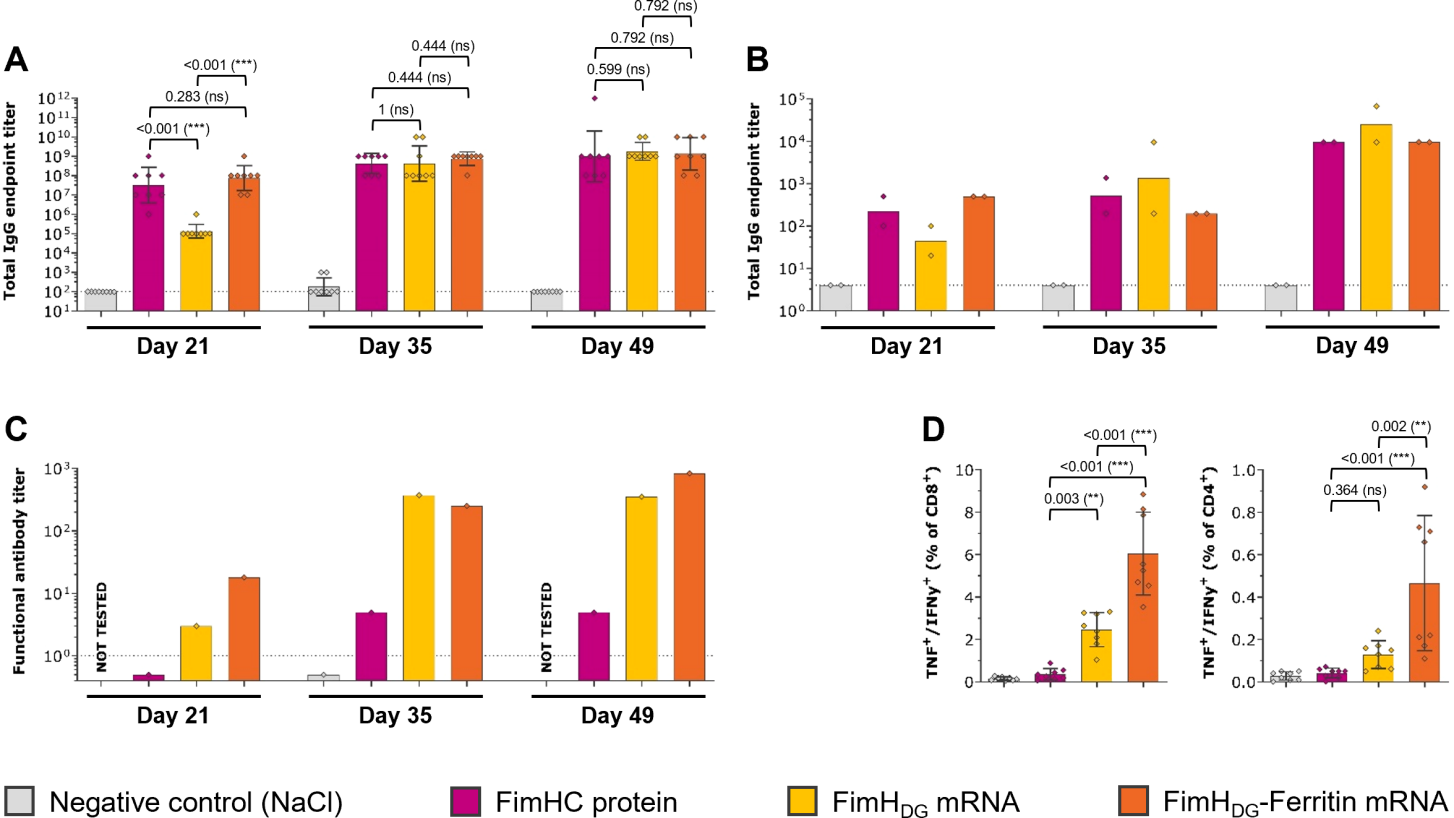
FimH-based mRNA vaccines containing unmodified nucleosides induced robust levels of both humoral and cellular immune responses in mice. Female BALB/c mice (n=8/group) were vaccinated IM three times on Day 0, 21, and 35 with 2 µg of PHAD-adjuvanted FimHC protein complex subunit vaccine (pink bars), 2 µg of FimH_DG_ mRNA vaccine (yellow bars), or 2 µg of FimH_DG_-Ferritin mRNA vaccine (orange bars). Mice receiving physiological saline (0.9% NaCl; grey bars) served as negative controls. FimH_L_-specific binding antibody endpoint titers in serum **(A)** or urine **(B)**, determined by ELISA, as well as functional serum antibody titers measured by BAI assay **(C)** in samples collected after one (Day 21), two (Day 35), or three (Day 49) vaccinations are displayed. Multifunctional TNF/IFN-γ-positive CD4^+^ and CD8^+^ T cells **(D)** were analyzed in splenocytes isolated on Day 49 by stimulation with FimH-specific peptides followed by intracellular cytokine staining and detection by flow cytometry. The gating strategy for this analysis is shown in **Supplementary Figure S2**. In **(A, D)**, diamond symbols represent individual animals, and bars depict either geometric means with geometric SD in **(A)** or means with SD in **(D)**. Each bar shown in **(B)** represents the geometric mean of two urine pools per group, each containing urine from four animals. Each bar depicted in **(C)** represents one functional antibody titer per group, determined based on pooled serum samples derived from all animals in the respective group. Dotted lines indicate the lower limit of quantification (LLOQ). Values below the LLOQ were set to 0.5 (non-responders in BAI assay; **C**). Data from individual days displayed in **(D)** were statistically analyzed using an ANOVA followed by the LSD post test. *P* values are displayed and significant differences between vaccines are marked by asterisks (**P < 0.01, ***P < 0.001; ns, not significant).

In order to assess the immunogenicity of the mRNA-based FimH vaccine candidates, BALB/cAnNRi mice received three IM injections of either FimH_DG_ or FimH_DG_-Ferritin mRNA vaccines, both containing unmodified nucleosides, or PHAD-adjuvanted FimHC protein complex subunit vaccine as a control. Animals receiving the FimH_DG_-Ferritin mRNA vaccine or the PHAD-adjuvanted FimHC protein subunit vaccine showed the highest levels of FimH_L_-specific IgG binding antibodies in serum (**Figure 2A**) and urine (**Figure 2B**) after the first vaccine administration (Day 21). Differences between the vaccine groups were less pronounced after the second or third vaccine administration. Functional antibody responses (i.e. antibodies preventing UPEC binding to target cells), as measured by BAI assay, were highest in the group immunized with the FimH_DG_-Ferritin mRNA vaccine (**Figure 2C**). In contrast to the binding antibody levels, the functional antibody responses measured in mice that had received the PHAD-adjuvanted FimHC protein subunit vaccine were substantially lower than in the mRNA vaccine-administered groups. In addition to humoral responses, the FimH_DG_ and FimH_DG_-Ferritin mRNA vaccines induced cellular responses. Significantly higher levels of FimH-specific CD4^+^ and CD8^+^ T cells producing IFN-γ and TNF were detected in the spleens of mice administered with FimH_DG_-Ferritin compared with FimH_DG_ mRNA, while there were no measurable T cell responses for the PHAD-adjuvanted FimHC protein subunit vaccine (**Figure 2D**; **Supplementary Figure S2**).

### 3.3 FimH_DG_ and FimH_DG_-Ferritin mRNA vaccine candidates induce robust levels of binding and functional antibodies in rats

**Figure 3:**
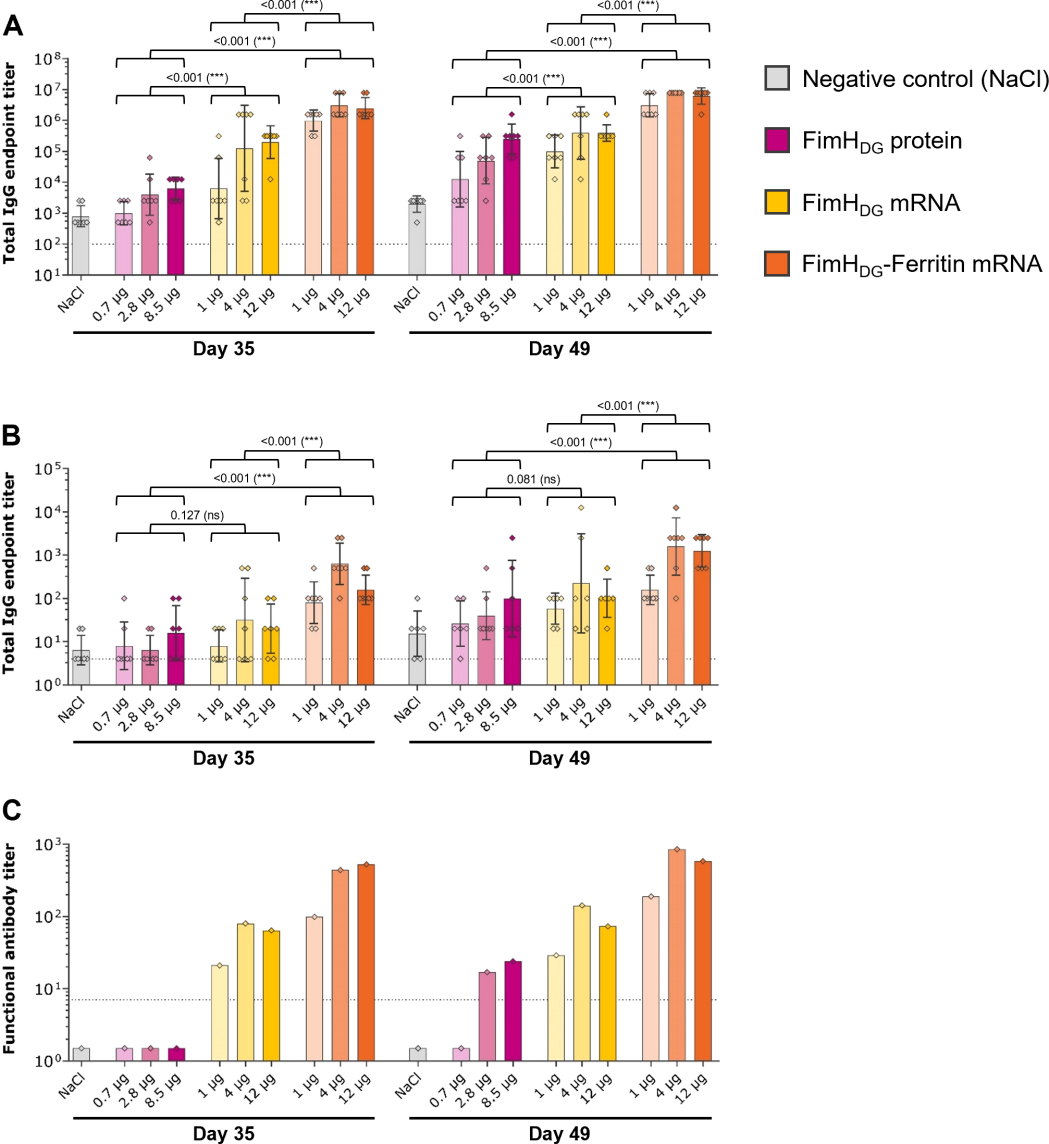
FimH-based mRNA vaccines using unmodified nucleosides induced robust levels of binding and functional antibody responses in rats. Female Wistar rats (first rat study; n=7/group) were vaccinated IM three times on Day 0, Day 21, and Day 35 with different doses of AS01-adjuvanted FimH_DG_ protein subunit vaccine (pink bars), FimH_DG_ mRNA vaccine (yellow bars), or FimH_DG_-Ferritin mRNA vaccine (orange bars) as indicated. Rats receiving physiological saline (0.9% NaCl; grey bars) served as negative controls. FimH_L_-specific binding antibody endpoint titers in serum **(A)** or urine **(B)**, determined by ELISA, as well as functional serum antibody titers measured by BAI assay **(C)** in samples collected after two (Day 35) or three (Day 49) vaccinations are displayed. In **(A, B)**, diamond symbols represent individual animals, and bars depict geometric means with geometric SD. Each bar depicted in **(C)** represents one functional antibody titer per group, determined based on pooled serum samples derived from all animals in the respective group. Dotted lines indicate the lower limit of quantification (LLOQ). Values below the LLOQ were set to 1.5 (non-responders in BAI assay; **C**). Individual days were statistically analyzed using an ANOVA followed by the LSD post test. *P* values are displayed and significant differences between vaccines across the doses tested are marked by asterisks (***P < 0.001; ns, not significant).

In the first confirmatory immunogenicity study in Wistar rats, various doses of the selected mRNA vaccine candidates FimH_DG_ and FimH_DG_-Ferritin containing unmodified nucleosides were tested, along with an AS01-adjuvanted FimH_DG_ protein subunit vaccine used as comparator (**Figure 3**). At the tested dosages, the FimH_L_-specific binding antibody levels in serum (**Figure 3A**) corresponded with the functional antibody responses, detected via the BAI assay (**Figure 3C**). Results indicate that the highest antibody titers were induced after the second and third administrations of the FimH_DG_-Ferritin mRNA vaccine candidate, followed by the FimH_DG_ mRNA vaccine and the protein subunit vaccine control. In general, the groups with the highest binding antibody titers in serum also had the highest titers in urine (**Figure 3B**), as well as the highest functional antibody levels in serum.

Together with the findings from the mouse study, these results clearly demonstrated that multimerization of the FimH antigen via fusion to ferritin enhanced the immunological potency compared to the monomeric mRNA design.

### 3.4 FimH_DG_-Ferritin mRNA vaccine including N1mΨ-modified nucleosides induces robust levels of binding and functional antibody responses in rats

Using modified nucleosides in vaccines, such as N1mΨ, has been shown to be immunogenic and efficacious as well as safe and well tolerated in humans, as evidenced for the two licensed COVID-19 mRNA vaccines, Spikevax^®^ (Moderna) and Comirnaty^®^ (Pfizer-BioNTech) (47, 48). This suggests that a wider therapeutic window (i.e., higher dose level resulting in higher immune responses and efficacy, associated with acceptable reactogenicity) can be achieved with modified-nucleoside mRNA as compared with unmodified-nucleoside mRNA vaccines (47–53).

Therefore, the *in vitro* immunostimulatory activity and *in vivo* immunogenicity of the lead candidate antigen design FimH_DG_-Ferritin were evaluated comparing unmodified or N1mΨ nucleoside containing mRNA vaccines. hPBMCs stimulated with the FimH_DG_-Ferritin mRNA vaccine containing unmodified nucleosides showed detectable levels of IFN-α. In contrast, the FimH_DG_-Ferritin mRNA vaccine containing N1mΨ did not induce detectable levels of IFN-α (**Supplementary Figure S3**).

**Figure 4:**
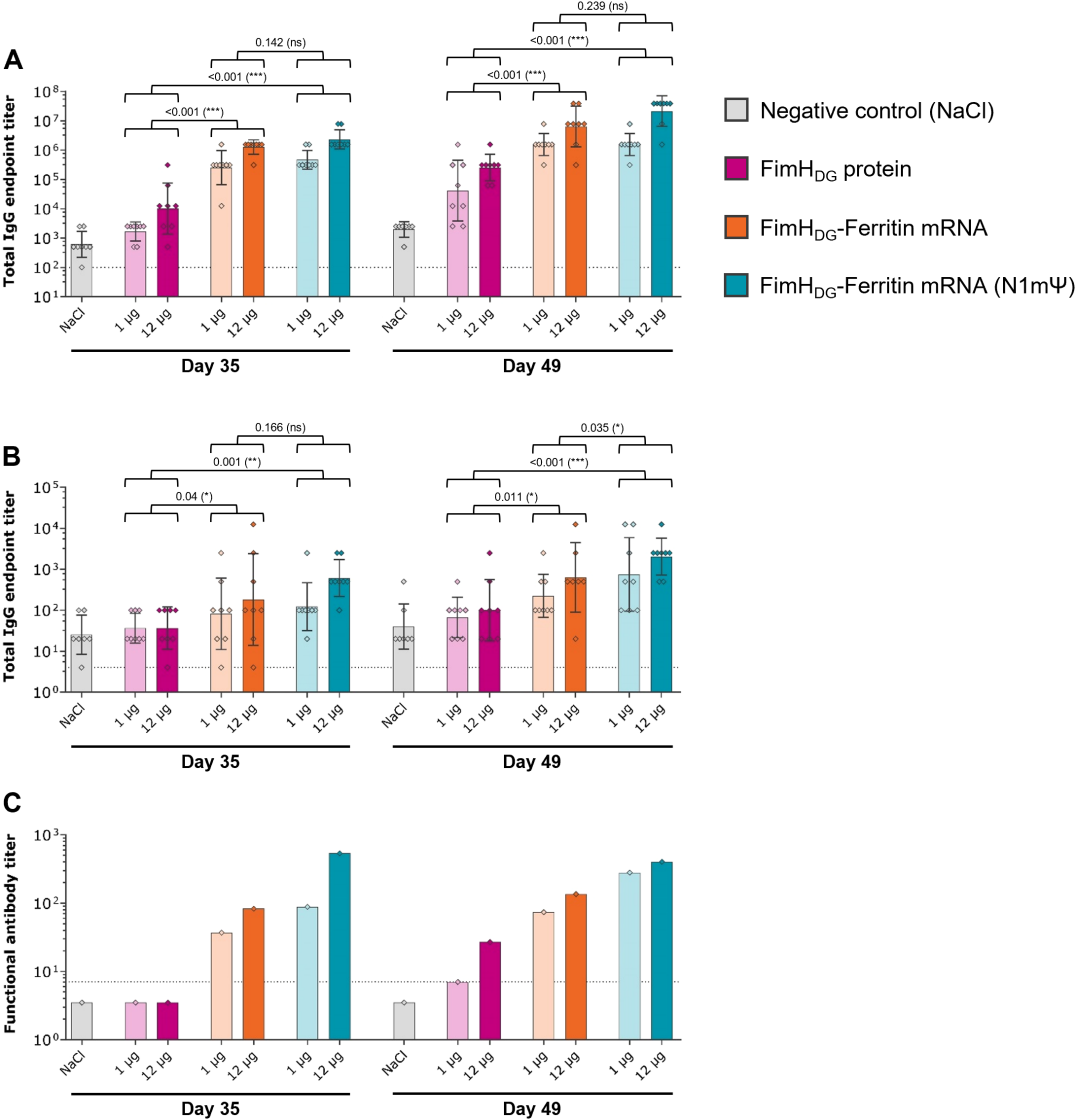
An mRNA vaccine using N1mΨ-modified nucleosides encoding FimH_DG_-Ferritin induced robust levels of binding and functional antibody responses in rats. Female Wistar rats (second rat study; n=8/group) were vaccinated IM three times on Day 0, Day 21, and Day 35 with different doses of AS01-adjuvanted FimH_DG_ protein subunit vaccine (pink bars), FimH_DG_-Ferritin mRNA vaccine containing unmodified nucleosides (orange bars), or FimH_DG_-Ferritin mRNA vaccine containing N1mΨ-modified nucleosides (blue bars) as indicated. Rats receiving physiological saline (0.9% NaCl; grey bars) served as negative controls. FimH_L_-specific binding antibody endpoint titers in serum **(A)** or urine **(B)**, determined by ELISA, as well as functional serum antibody titers measured by BAI assay **(C)** in samples collected after two (Day 35) or three (Day 49) vaccinations are displayed. In **(A)** and **(B)**, diamond symbols represent individual animals, and bars depict geometric means with geometric SD. Each bar depicted in **(C)** represents one functional antibody titer per group, determined based on pooled serum samples derived from all animals in the respective group. Dotted lines indicate the lower limit of quantification (LLOQ). Values below the LLOQ were set 3.5 (non-responders in BAI assay; **C**). Individual days were statistically analyzed using an ANOVA followed by the LSD post test. *P* values are displayed and significant differences between vaccines across the doses tested are marked by asterisks (*P < 0.05, **P < 0.01, ***P < 0.001; ns, not significant).

In the second study with Wistar rats, similar FimH_L_-specific serum IgG levels were induced by the FimH_DG_-Ferritin mRNA vaccines containing unmodified and N1mΨ-modified nucleosides upon IM injection, while the tested dosages of AS01-adjuvanted FimH_DG_ protein subunit vaccine used as a comparator induced significantly lower binding antibody responses (**Figure 4A**). Among the tested mRNA vaccine candidates, FimH_DG_-Ferritin with N1mΨ induced significantly higher IgG levels in the urine after three administrations (**Figure 4B**) and the highest functional antibody titers in serum (**Figure 4C**).

## 4 Discussion

Data from these proof-of-concept immunization studies in mice and rats using FimH_DG_ mRNA vaccine candidates with unmodified nucleosides demonstrated the induction of significant levels of FimH-specific immune responses in both species. Multimeric presentation of vaccine-candidate antigens has been shown to enhance uptake and delivery by antigen-presenting cells, driving B-cell activation through receptor cross-linking to establish a protective humoral response (30, 54, 55). Consistently, we found that the FimH_DG_-Ferritin mRNA-based candidate vaccine was more immunogenic compared to the FimH_DG_ vaccine, indicating that the multimeric presentation of the FimH antigen on a PNP surface enhanced the immunogenicity compared to a monomeric antigen design. The improved immunogenicity of FimH_DG_ when fused to ferritin may also be derived from the intrinsic stability of ferritin nanoparticles, adding to the growing literature demonstrating the utility and versatility of ferritin-based vaccines (56).

Different vaccine platforms have been harnessed in the development of a vaccine against rUTI. In a Phase 1/2 study, a bioconjugate vaccine including the four most relevant O-antigens expressed in *E. coli* as glycoproteins induced carbohydrate-specific opsonophagocytic antibodies but failed to reduce the frequency of UTI due to the serotypes expressing the targeted O-antigens (57). In non-human primates, a vaccine that combines FimH and O-antigens reduced bacterial load of UTI (58). Hence, there is a need for a vaccine that induces robust transudation of anti-adhesive antibodies into the human bladder. Transudation of antibodies to the urogenital tract has been demonstrated following administration of the human papillomavirus (HPV) vaccine (59) and antibody presence in urine (60).

Whilst we observed a strong IgG response in serum following administration of FimH and FimH_DG_-Ferritin vaccine candidates, urine IgG levels were relatively low in these animals, possibly due to the absence of bacterial infection. This could be linked to a hypothesized mode of action for a FimH-based vaccine, whereby serum-derived IgG could provide protection against UPEC in the urinary tract upon increased blood vessel permeability due to the inflammation induced by the bacterial infection. Since a relatively low number of UPEC bacteria initiate a UTI (61), asymptomatic inflammation and increased blood vessel permeability may allow serum-derived anti-FimH IgG antibodies to transudate into the bladder and block further binding of UPEC to uroepithelial cells, whilst unbound bacteria are eliminated through urinary flow. If this hypothesis is correct, FimH-specific functional antibodies in serum could act as a correlate of protection.

In addition to a strong humoral response, our FimH_DG_-Ferritin mRNA vaccine induced splenic T cell responses that were significantly higher than those observed with the protein subunit vaccine control at the tested dosages. The ability of mRNA vaccines to elicit more robust CD8^+^ T cell responses compared with protein subunit vaccines has been described previously (62–64). Since T cell responses are an important feature of natural immunity and susceptibility to UPEC infection (65), a strong, specific T cell response may enhance vaccine efficacy. Data presented here demonstrate the induction of multifunctional TNF/IFN-γ-positive CD4^+^ and CD8^+^ T cells upon administration of mRNA vaccines. These cells are indicative of a T helper type 1 (Th1) response which is crucial for bacteria clearance in the bladder (65). The mRNA vaccine technology used in this study has been previously shown to induce enrichment of lung-resident memory CD8+ T cells in mice (66). Mucosal memory T cells, which reside within peripheral tissues, are able to protect against recurrent UPEC infections in mice depleted of systemic T cells (67). In addition, tissue-resident memory T cells (T_RM_) cells can rapidly respond to an infection without needing to migrate, proliferate or differentiate, and can contribute to improved vaccine efficacy (68, 69). Indeed, previous studies have shown that vaccines designed to generate pathogen-specific T_RM_ populations in mucosal tissues could provide long-lasting protection and reduce recurrence at mucosal surfaces (69, 70). Future research could explore the most effective strategies for inducing robust T cell responses that are most effective in driving bacterial clearance in the bladder, such as through mucosal vaccination.

Nucleoside modification of mRNA (such as addition of pseudouridine) suppresses the immunostimulatory effect of RNA (71, 72) and increases its translation in therapeutic applications (72, 73). In the current study, nucleoside modification (N1mΨ) in the FimH_DG_-Ferritin candidate vaccine improved binding and functional antibody responses in female rats and also led to reduced induction of IFN-α in hPBMCs, with the potential to translate to a wider range of tolerable vaccine doses in humans. Overall, the data generated in these experiments demonstrate that FimH_DG_-Ferritin mRNA-based candidate containing modified nucleosides is a promising UPEC vaccine for further development. Key to this development is the assessment of protective efficacy following FimH_DG_-Ferritin mRNA vaccine administration in an *in vivo* model of UTI, which will be evaluated in future studies.

The studies reported here do have some limitations. Whilst we employed protein subunit vaccines as a control, direct comparison of doses between mRNA and protein vaccines are difficult since the systems employed prevent direct determination of protein levels expressed from mRNA *in vivo*. Given the high prevalence of rUTI in women, studies were performed with female animals only; however, future studies with male rodents would be beneficial. In addition, statistical analyses were not performed for all experiments due to pooling of collected samples, which limits assessment of functional antibody differences between groups. As such, future studies will be designed without pooling samples. Nonetheless, the methods reported here have demonstrated good reproducibility, with comparable titers of functional antibodies and serum and urine IgG seen across all three animal studies with the unmodified FimH_DG_-Ferritin mRNA vaccine.

## 5 Conclusion

With the success of mRNA vaccines against viral infections, the development of mRNA vaccines against bacterial pathogens is still in the early stages, with only three vaccine candidates in clinical trials (74). Our mRNA-based vaccine FimH_DG_-Ferritin with N1mΨ-modified nucleosides is a promising candidate for further development as a vaccine against UPEC, a leading cause of rUTI. As there is already significant clinical data on the safety profile of mRNA vaccines, and a well-established route for regulatory approval (36, 75), future research will focus on further characterization of the FimH_DG_-Ferritin vaccine, particularly in terms of the protective efficacy in animal models, with the aim of progressing into clinical development.

## Data Availability

The original contributions presented in the study are included in the article/**Supplementary Material**. Further inquiries can be directed to the corresponding author.

## References

[1] Yang X Chen H Zheng Y Qu S Wang H Yi F. Disease burden and long-term trends of urinary tract infections: A worldwide report. Front Public Health. (2022) 10:888205. doi: 10.3389/fpubh.2022.888205, PMID: 35968451 PMC9363895

[2] Houston CG Azar WS Huang SS Rubin R Dorris CS Sussman RD. A cost savings analysis of topical estrogen therapy in urinary tract infection prevention among postmenopausal women. Urol Pract. (2024) 11:257–66. doi: 10.1097/upj.0000000000000513, PMID: 38154005

[3] Brumbaugh AR Mobley HL. Preventing urinary tract infection: Progress toward an effective *Escherichia coli* vaccine. Expert Rev Vaccines. (2012) 11:663–76. doi: 10.1586/erv.12.36, PMID: 22873125 PMC3498450

[4] Medina M Castillo-Pino E. An introduction to the epidemiology and burden of urinary tract infections. Ther Adv Urol. (2019) 11:1756287219832172. doi: 10.1177/1756287219832172, PMID: 31105774 PMC6502976

[5] Kwok M McGeorge S Mayer-Coverdale J Graves B Paterson DL Harris PNA. Guideline of guidelines: Management of recurrent urinary tract infections in women. BJU Int. (2022) 130:11–22. doi: 10.1111/bju.15756, PMID: 35579121 PMC9790742

[6] Aggarwal N Leslie SW. Recurrent urinary tract infections. In: StatPearls. StatPearls Publishing, Treasure Island (FL (2025). 32491411

[7] Flores-Mireles AL Walker JN Caparon M Hultgren SJ. Urinary tract infections: epidemiology, mechanisms of infection and treatment options. Nat Rev Microbiol. (2015) 13:269–84. doi: 10.1038/nrmicro3432, PMID: 25853778 PMC4457377

[8] Hospenthal MK Waksman G. The remarkable biomechanical properties of the type 1 chaperone-usher pilus: A structural and molecular perspective. Microbiol Spectr. (2019) 7. doi: 10.1128/microbiolspec.psib-0010-2018, PMID: 30681068 PMC11588285

[9] Delcaru C Alexandru I Podgoreanu P Grosu M Stavropoulos E Chifiriuc MC. Microbial biofilms in urinary tract infections and prostatitis: etiology, pathogenicity, and combating strategies. Pathogens. (2016) 5:65. doi: 10.3390/pathogens5040065, PMID: 27916925 PMC5198165

[10] Naziri Z Kilegolan JA Moezzi MS Derakhshandeh A. Biofilm formation by uropathogenic *Escherichia coli*: A complicating factor for treatment and recurrence of urinary tract infections. J Hosp Infect. (2021) 117:9–16. doi: 10.1016/j.jhin.2021.08.017, PMID: 34428502

[11] Tarlton NJ Moritz C Adams-Sapper S Riley LW. Genotypic analysis of uropathogenic *Escherichia coli* to understand factors that impact the prevalence of β-lactam-resistant urinary tract infections in a community. J Glob Antimicrob Resist. (2019) 19:173–80. doi: 10.1016/j.jgar.2019.03.002, PMID: 30872040 PMC6739196

[12] Bradley MS Cabrera C Clark SG Sassani J Venuti K Ackenbom MF. Sporadic compared to recurrent urinary tract infections: Considerations for urogynecologic patients. Neurourol Urodyn. (2020) 39:2186–91. doi: 10.1002/nau.24471, PMID: 32803912 PMC7709465

[13] Bauer HW Alloussi S Egger G Blümlein HM Cozma G Schulman CC. A long-term, multicenter, double-blind study of an *Escherichia coli* extract (OM-89) in female patients with recurrent urinary tract infections. Eur Urol. (2005) 47:542–8. doi: 10.1016/j.eururo.2004.12.009, PMID: 15774256

[14] Hopkins WJ Elkahwaji J Beierle LM Leverson GE Uehling DT. Vaginal mucosal vaccine for recurrent urinary tract infections in women: Results of a phase 2 clinical trial. J Urol. (2007) 177:1349–53. doi: 10.1016/j.juro.2006.11.093, PMID: 17382730

[15] Benito-Villalvilla C Cirauqui C Diez-Rivero CM Casanovas M Subiza JL Palomares O. MV140, a sublingual polyvalent bacterial preparation to treat recurrent urinary tract infections, licenses human dendritic cells for generating Th1, Th17, and IL-10 responses via Syk and MyD88. Mucosal Immunol. (2017) 10:924–35. doi: 10.1038/mi.2016.112, PMID: 27966556

[16] Mak Q Greig J Dasgupta P Malde S Raison N. Bacterial vaccines for the management of recurrent urinary tract infections: A systematic review and meta-analysis. Eur Urol Focus. (2024) 10:761–9. doi: 10.1016/j.euf.2024.04.002, PMID: 38644097

[17] FDA. Vaccines Licensed for Use in the United States (2025). Available online at: https://www.fda.gov/vaccines-blood-biologics/vaccines/vaccines-licensed-use-united-states (Accessed June 27, 2025).

[18] Sarshar M Behzadi P Ambrosi C Zagaglia C Palamara AT Scribano D. FimH and anti-adhesive therapeutics: A disarming strategy against uropathogens. Antibiotics (Basel). (2020) 9:397. doi: 10.3390/antibiotics9070397, PMID: 32664222 PMC7400442

[19] Mirzahosseini HK Najmeddin F Najafi A Ahmadi A Sharifnia H Khaledi A. Correlation of biofilm formation, virulence factors, and phylogenetic groups among *Escherichia coli* strains causing urinary tract infection: A global systematic review and meta-analysis. J Res Med Sci. (2023) 28:66. doi: 10.4103/jrms.jrms_637_22, PMID: 38024522 PMC10668210

[20] Hung C-S Bouckaert J Hung D Pinkner J Widberg C DeFusco A. Structural basis of tropism of *Escherichia coli* to the bladder during urinary tract infection. Mol Microbiol. (2002) 44:903–15. doi: 10.1046/j.1365-2958.2002.02915.x, PMID: 12010488

[21] Sauer MM Jakob RP Eras J Baday S Eriş D Navarra G. Catch-bond mechanism of the bacterial adhesin FimH. Nat Commun. (2016) 7:10738. doi: 10.1038/ncomms10738, PMID: 26948702 PMC4786642

[22] Kalas V Pinkner JS Hannan TJ Hibbing ME Dodson KW Holehouse AS. Evolutionary fine-tuning of conformational ensembles in FimH during host-pathogen interactions. Sci Adv. (2017) 3:e1601944. doi: 10.1126/sciadv.1601944, PMID: 28246638 PMC5302871

[23] Rabbani S Fiege B Eris D Silbermann M Jakob RP Navarra G. Conformational switch of the bacterial adhesin FimH in the absence of the regulatory domain: Engineering a minimalistic allosteric system. J Biol Chem. (2018) 293:1835–49. doi: 10.1074/jbc.M117.802942, PMID: 29180452 PMC5798311

[24] Silmon de Monerri NC Che Y Lees JA Jasti J Wu H Griffor MC. Structure-based design of an immunogenic, conformationally stabilized FimH antigen for a urinary tract infection vaccine. PloS Pathog. (2025) 21:e1012325. doi: 10.1371/journal.ppat.1012325, PMID: 39970181 PMC12136410

[25] López-Sagaseta J Malito E Rappuoli R Bottomley MJ. Self-assembling protein nanoparticles in the design of vaccines. Comput Struct Biotechnol J. (2016) 14:58–68. doi: 10.1016/j.csbj.2015.11.001, PMID: 26862374 PMC4706605

[26] Joyce MG Chen WH Sankhala RS Hajduczki A Thomas PV Choe M. SARS-CoV-2 ferritin nanoparticle vaccines elicit broad SARS coronavirus immunogenicity. Cell Rep. (2021) 37:110143. doi: 10.1016/j.celrep.2021.110143, PMID: 34919799 PMC8651551

[27] Song N Zhang J Zhai J Hong J Yuan C Liang M. Ferritin: A multifunctional nanoplatform for biological detection, imaging diagnosis, and drug delivery. Acc Chem Res. (2021) 54:3313–25. doi: 10.1021/acs.accounts.1c00267, PMID: 34415728

[28] Yang F Mariz FC Zhao X Spagnoli G Ottonello S Müller M. Broad neutralization responses against oncogenic human papillomaviruses induced by a minor capsid L2 polytope genetically incorporated into bacterial ferritin nanoparticles. Front Immunol. (2020) 11:606569. doi: 10.3389/fimmu.2020.606569, PMID: 33343580 PMC7746619

[29] Yin S Davey K Dai S Liu Y Bi J. A critical review of ferritin as a drug nanocarrier: Structure, properties, comparative advantages and challenges. Particuology. (2022) 64:65–84. doi: 10.1016/j.partic.2021.04.020

[30] Corbett KS Moin SM Yassine HM Cagigi A Kanekiyo M Boyoglu-Barnum S. Design of nanoparticulate group 2 influenza virus hemagglutinin stem antigens that activate unmutated ancestor B cell receptors of broadly neutralizing antibody lineages. mBio. (2019) 10:e02810–18. doi: 10.1128/mbio.02810-18, PMID: 30808695 PMC6391921

[31] Fan K Jiang B Guan Z He J Yang D Xie N. Fenobody: A ferritin-displayed nanobody with high apparent affinity and half-life extension. Anal Chem. (2018) 90:5671–7. doi: 10.1021/acs.analchem.7b05217, PMID: 29634235

[32] Yassine HM Boyington JC McTamney PM Wei C-J Kanekiyo M Kong W-P. Hemagglutinin-stem nanoparticles generate heterosubtypic influenza protection. Nat Med. (2015) 21:1065–70. doi: 10.1038/nm.3927, PMID: 26301691

[33] Kanekiyo M Wei C-J Yassine HM McTamney PM Boyington JC Whittle JRR. Self-assembling influenza nanoparticle vaccines elicit broadly neutralizing H1N1 antibodies. Nature. (2013) 499:102–6. doi: 10.1038/nature12202, PMID: 23698367 PMC8312026

[34] Sun W He L Zhang H Tian X Bai Z Sun L. The self-assembled nanoparticle-based trimeric RBD mRNA vaccine elicits robust and durable protective immunity against SARS-CoV-2 in mice. Signal Transduct Target Ther. (2021) 6:340. doi: 10.1038/s41392-021-00750-w, PMID: 34504054 PMC8426336

[35] Mu Z Wiehe K Saunders KO Henderson R Cain DW Parks R. mRNA-encoded HIV-1 Env trimer ferritin nanoparticles induce monoclonal antibodies that neutralize heterologous HIV-1 isolates in mice. Cell Rep. (2022) 38:110514. doi: 10.1016/j.celrep.2022.110514, PMID: 35294883 PMC8922439

[36] Gote V Bolla PK Kommineni N Butreddy A Nukala PK Palakurthi SS. A comprehensive review of mRNA vaccines. Int J Mol Sci. (2023) 24:2700. doi: 10.3390/ijms24032700, PMID: 36769023 PMC9917162

[37] Whelan S Lucey B Finn K. Uropathogenic *Escherichia coli* (UPEC)-associated urinary tract infections: The molecular basis for challenges to effective treatment. Microorganisms. (2023) 11:2169. doi: 10.3390/microorganisms11092169, PMID: 37764013 PMC10537683

[38] Eldridge GR Hughey H Rosenberger L Martin SM Shapiro AM D'Antonio E. Safety and immunogenicity of an adjuvanted *Escherichia coli* adhesin vaccine in healthy women with and without histories of recurrent urinary tract infections: Results from a first-in-human phase 1 study. Hum Vaccin Immunother. (2021) 17:1262–70. doi: 10.1080/21645515.2020.1834807, PMID: 33325785 PMC8078672

[39] Adamo R Grosse HW Petsch B Phogat S Rauch S Roier S. inventors; nucleic acid based vaccine encoding an *escherichia coli* fimH antigenic polypeptide international patent application PCT/EP2023/063799. (2023).

[40] Eldridge G Martin SM. Compositions of vaccines and adjuvants and methods for the treatment of urinary tract infections. United States of America (2015). Patent Application US20150086591.

[41] Percie du Sert N Hurst V Ahluwalia A Alam S Avey MT Baker M. The ARRIVE guidelines 2.0: Updated guidelines for reporting animal research. BMC Vet Res. (2020) 16:242. doi: 10.1186/s12917-020-02451-y, PMID: 32660541 PMC7359286

[42] Rauch S Jasny E Schmidt KE Petsch B. New vaccine technologies to combat outbreak situations. Front Immunol. (2018) 9:1963. doi: 10.3389/fimmu.2018.01963, PMID: 30283434 PMC6156540

[43] Iavarone C O’Hagan DT Yu D Delahaye NF Ulmer JB. Mechanism of action of mRNA-based vaccines. Expert Rev Vaccines. (2017) 16:871–81. doi: 10.1080/14760584.2017.1355245, PMID: 28701102

[44] Andersen TE Khandige S Madelung M Brewer J Kolmos HJ Møller-Jensen J. *Escherichia coli* uropathogenesis *in vitro*: Invasion, cellular escape, and secondary infection analyzed in a human bladder cell infection model. Infect Immun. (2012) 80:1858–67. doi: 10.1128/iai.06075-11, PMID: 22354025 PMC3347433

[45] Laemmli UK. Cleavage of structural proteins during the assembly of the head of bacteriophage T4. Nature. (1970) 227:680–5. doi: 10.1038/227680a0, PMID: 5432063

[46] Lee NK Cho S Kim I-S. Ferritin – a multifaceted protein scaffold for biotherapeutics. Exp Mol Med. (2022) 54:1652–7. doi: 10.1038/s12276-022-00859-0, PMID: 36192487 PMC9527718

[47] Baden L R El Sahly Hana M Essink B Kotloff K Frey S Novak R. Efficacy and safety of the mRNA-1273 SARS-CoV-2 vaccine. N Engl J Med. (2021) 384:403–16. doi: 10.1056/NEJMoa2035389, PMID: 33378609 PMC7787219

[48] Polack F P Thomas Stephen J Kitchin N Absalon J Gurtman A Lockhart S. Safety and efficacy of the BNT162b2 mRNA Covid-19 vaccine. N Engl J Med. (2020) 383:2603–15. doi: 10.1056/NEJMoa2034577, PMID: 33301246 PMC7745181

[49] Anderson BR Muramatsu H Jha BK Silverman RH Weissman D Karikó K. Nucleoside modifications in RNA limit activation of 2′-5′-oligoadenylate synthetase and increase resistance to cleavage by RNase L. Nucleic Acids Res. (2011) 39:9329–38. doi: 10.1093/nar/gkr586, PMID: 21813458 PMC3241635

[50] Kremsner PG Mann P Kroidl A Leroux-Roels I Schindler C Gabor JJ. Safety and immunogenicity of an mRNA-lipid nanoparticle vaccine candidate against SARS-CoV-2. Wien Klin Wochenschr. (2021) 133:931–41. doi: 10.1007/s00508-021-01922-y, PMID: 34378087 PMC8354521

[51] Stuart L M. In gratitude for mRNA vaccines. N Engl J Med. (2021) 385:1436–8. doi: 10.1056/NEJMcibr2111445, PMID: 34569728

[52] Thomas S J Moreira Edson D Kitchin N Absalon J Gurtman A Lockhart S. Safety and efficacy of the BNT162b2 mRNA Covid-19 vaccine through 6 months. N Engl J Med. (2021) 385:1761–73. doi: 10.1056/NEJMoa2110345, PMID: 34525277 PMC8461570

[53] Walsh E E Frenck Robert W Falsey Ann R Kitchin N Absalon J Gurtman A. Safety and immunogenicity of two RNA-based Covid-19 vaccine candidates. N Engl J Med. (2020) 383:2439–50. doi: 10.1056/NEJMoa2027906, PMID: 33053279 PMC7583697

[54] Zepeda-Cervantes J Ramírez-Jarquín JO Vaca L. Interaction between virus-like particles (VLPs) and pattern recognition receptors (PRRs) from dendritic cells (DCs): Toward better engineering of VLPs. Front Immunol. (2020) 11:1100. doi: 10.3389/fimmu.2020.01100, PMID: 32582186 PMC7297083

[55] Kelly HG Tan HX Juno JA Esterbauer R Ju Y Jiang W. Self-assembling influenza nanoparticle vaccines drive extended germinal center activity and memory B cell maturation. JCI Insight. (2020) 5. doi: 10.1172/jci.insight.136653, PMID: 32434990 PMC7259527

[56] Cao S Ma D Ji S Zhou M Zhu S. Self-assembled ferritin nanoparticles for delivery of antigens and development of vaccines: From structure and property to applications. Molecules. (2024) 29. doi: 10.3390/molecules29174221, PMID: 39275069 PMC11397193

[57] Huttner A Hatz C van den Dobbelsteen G Abbanat D Hornacek A Frölich R. Safety, immunogenicity, and preliminary clinical efficacy of a vaccine against extraintestinal pathogenic *Escherichia coli* in women with a history of recurrent urinary tract infection: A randomised, single-blind, placebo-controlled phase 1b trial. Lancet Infect Dis. (2017) 17:528–37. doi: 10.1016/S1473-3099(17)30108-1, PMID: 28238601

[58] Chorro L Ciolino T Torres Caresse L Illenberger A Aglione J Corts P. A cynomolgus monkey E. coli urinary tract infection model confirms efficacy of new FimH vaccine candidates. Infect Immun. (2024) 92:e00169–24. doi: 10.1128/iai.00169-24, PMID: 39297649 PMC11475676

[59] Schwarz TF Kocken M Petäjä T Einstein MH Spaczynski M Louwers JA. Correlation between levels of human papillomavirus (HPV)-16 and 18 antibodies in serum and cervicovaginal secretions in girls and women vaccinated with the HPV-16/18 AS04-adjuvanted vaccine. Hum Vaccin. (2010) 6:1054–61. doi: 10.4161/hv.6.12.13399, PMID: 21157180

[60] Pattyn J Van Keer S Téblick L Van Damme P Vorsters A. Non-invasive assessment of vaccine-induced HPV antibodies via first-void urine. Front Immunol. (2020) 11:1657. doi: 10.3389/fimmu.2020.01657, PMID: 32849573 PMC7419594

[61] Stærk K Grønnemose RB Nielsen TK Petersen NA Palarasah Y Torres-Puig S. *Escherichia coli* type-1 fimbriae are critical to overcome initial bottlenecks of infection upon low-dose inoculation in a porcine model of cystitis. Microbiol (Reading). (2021) 167:1101. doi: 10.1099/mic.0.001101, PMID: 34623231 PMC8698211

[62] Hirai T Yoshioka Y. Considerations of CD8(+) T cells for optimized vaccine strategies against respiratory viruses. Front Immunol. (2022) 13:918611. doi: 10.3389/fimmu.2022.918611, PMID: 35774782 PMC9237416

[63] Park HJ Bang YJ Kwon SP Kwak W Park SI Roh G. Analyzing immune responses to varied mRNA and protein vaccine sequences. NPJ Vaccines. (2023) 8:84. doi: 10.1038/s41541-023-00684-0, PMID: 37271785 PMC10239716

[64] Wu Y Zhang H Meng L Li F Yu C. Comparison of immune responses elicited by SARS-CoV-2 mRNA and recombinant protein vaccine candidates. Front Immunol. (2022) 13:906457. doi: 10.3389/fimmu.2022.906457, PMID: 35663946 PMC9161160

[65] Wu J Abraham SN. The roles of T cells in bladder pathologies. Trends Immunol. (2021) 42:248–60. doi: 10.1016/j.it.2021.01.003, PMID: 33536141 PMC7914211

[66] Corleis B Hoffmann D Rauch S Fricke C Roth N Gergen J. Efficacy of an unmodified bivalent mRNA vaccine against SARS-CoV-2 variants in female small animal models. Nat Commun. (2023) 14:816. doi: 10.1038/s41467-023-36110-1, PMID: 36781853 PMC9924835

[67] Rousseau M Lacerda Mariano L Canton T Ingersoll MA. Tissue-resident memory T cells mediate mucosal immunity to recurrent urinary tract infection. Sci Immunol. (2023) 8:eabn4332. doi: 10.1126/sciimmunol.abn4332, PMID: 37235683

[68] Parga-Vidal L van Aalderen MC Stark R van Gisbergen KPJM. Tissue-resident memory T cells in the urogenital tract. Nat Rev Nephrol. (2022) 18:209–23. doi: 10.1038/s41581-021-00525-0, PMID: 35079143

[69] Xu G Li Y Lu G Xie D. Tissue-resident memory T cells in urinary tract diseases. Front Immunol. (2025) 16:1535930. doi: 10.3389/fimmu.2025.1535930, PMID: 40066439 PMC11891219

[70] Lacerda Mariano L Ingersoll MA. The immune response to infection in the bladder. Nat Rev Urol. (2020) 17:439–58. doi: 10.1038/s41585-020-0350-8, PMID: 32661333

[71] Karikó K Buckstein M Ni H Weissman D. Suppression of RNA recognition by Toll-like receptors: the impact of nucleoside modification and the evolutionary origin of RNA. Immunity. (2005) 23:165–75. doi: 10.1016/j.immuni.2005.06.008, PMID: 16111635

[72] Karikó K Muramatsu H Welsh FA Ludwig J Kato H Akira S. Incorporation of pseudouridine into mRNA yields superior nonimmunogenic vector with increased translational capacity and biological stability. Mol Ther. (2008) 16:1833–40. doi: 10.1038/mt.2008.200, PMID: 18797453 PMC2775451

[73] Bernard MC Bazin E Petiot N Lemdani K Commandeur S Verdelet C. The impact of nucleoside base modification in mRNA vaccine is influenced by the chemistry of its lipid nanoparticle delivery system. Mol Ther Nucleic Acids. (2023) 32:794–806. doi: 10.1016/j.omtn.2023.05.004, PMID: 37346973 PMC10280092

[74] Khlebnikova A Kirshina A Zakharova N Ivanov R Reshetnikov V. Current progress in the development of mRNA vaccines against bacterial infections. Int J Mol Sci. (2024) 25. doi: 10.3390/ijms252313139, PMID: 39684849 PMC11642352

[75] Bergstrom C Fischer NO Kubicek-Sutherland JZ Stromberg ZR. mRNA vaccine platforms to prevent bacterial infections. Trends Mol Med. (2024) 30:524–6. doi: 10.1016/j.molmed.2024.02.013, PMID: 38485647

