# Supplementary material for: An mRNA-based FimH nanoparticle vaccine against uropathogenic Escherichia coli is highly immunogenic in rodents
Source: Front Immunol. 2025 Nov 3;16:1668937. doi: 10.3389/fimmu.2025.1668937 (PMC12620438; doi:10.3389/fimmu.2025.1668937)
Supplement: Supplementary file 1 [file DataSheet1.docx]

Supplementary Material

## Supplementary Figures


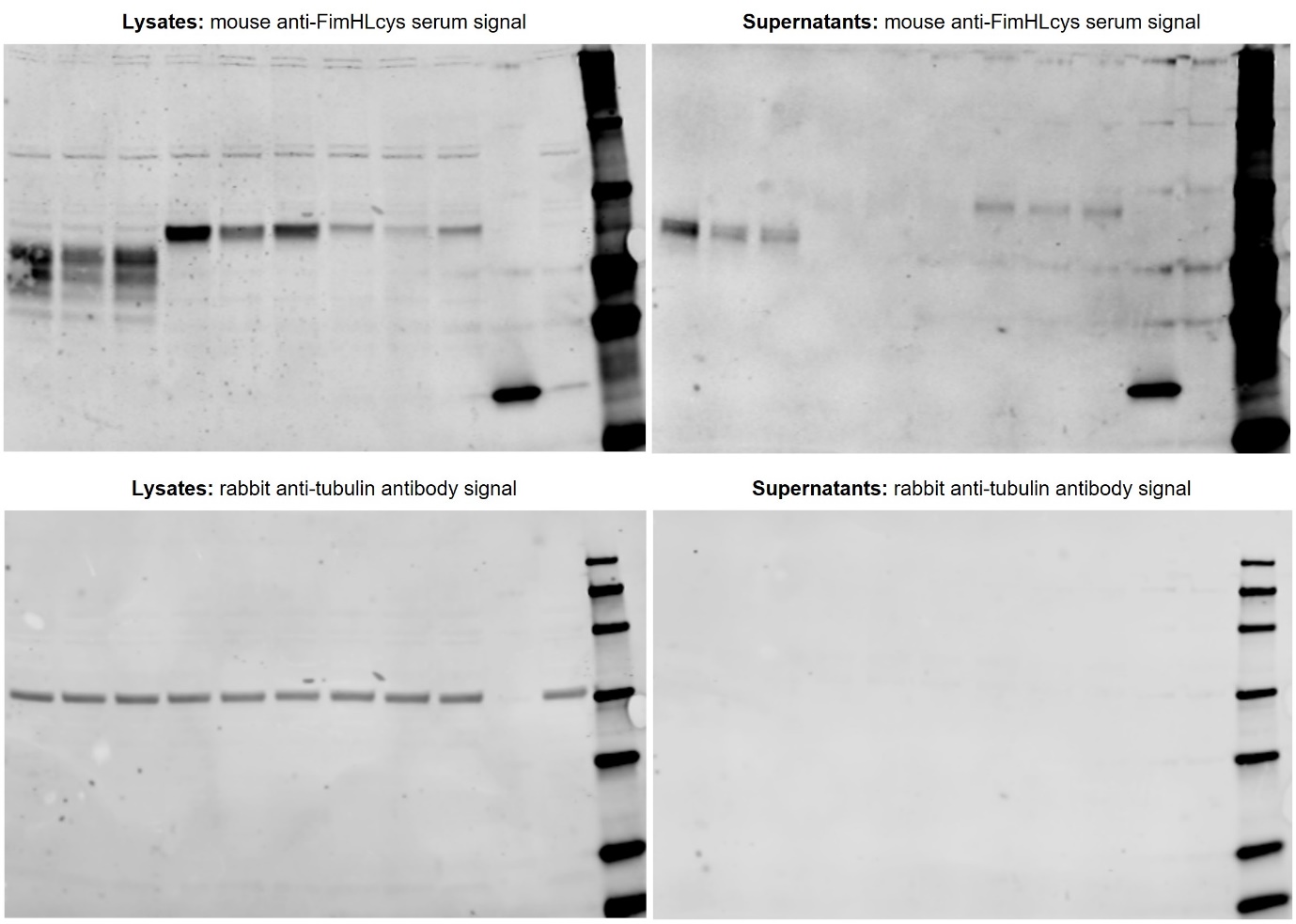


**Supplementary Figure 1. Western blot images of FimH expression in cell lysates and supernatants**.

Uncropped western blot images from Figure 1C of FimH expression in HEK 293T cell lysates and supernatants are shown. Details are described in Figure 1C.


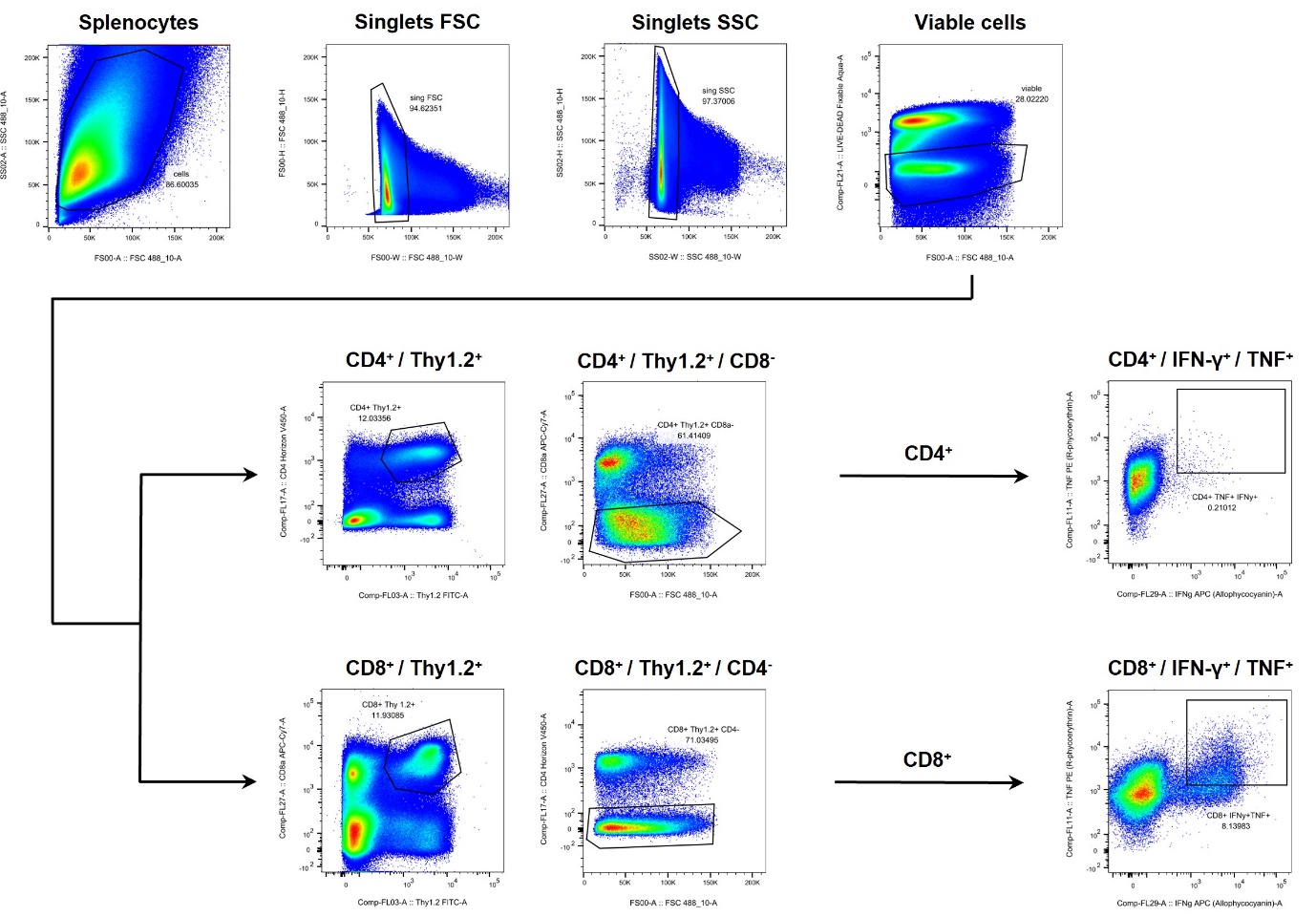


**Supplementary Figure 2. Gating strategy for T cell analysis.**

Female BALB/cAnNRj mice were vaccinated and splenocytes were isolated as described in Figure 2D. Multifunctional IFN-γ/TNF-positive CD4^+^ T and CD8^+^ T cells were analyzed in splenocytes stimulated with a FimH-specific peptide library followed by intracellular cytokine staining and detection by ﬂow cytometry. The flow blots shown illustrate the gating strategy using splenocytes isolated from a mouse vaccinated with FimH_DG_-Ferritin mRNA vaccine as an example. T cells were characterized as singlets, viable cells, Thy1.2^+^/CD4^+^ /CD8^-^ or Thy1.2^+^/CD8^+^ /CD4^-^ and subdivided into multifunctional T cell subsets based on their expression of IFN-γ and TNF.


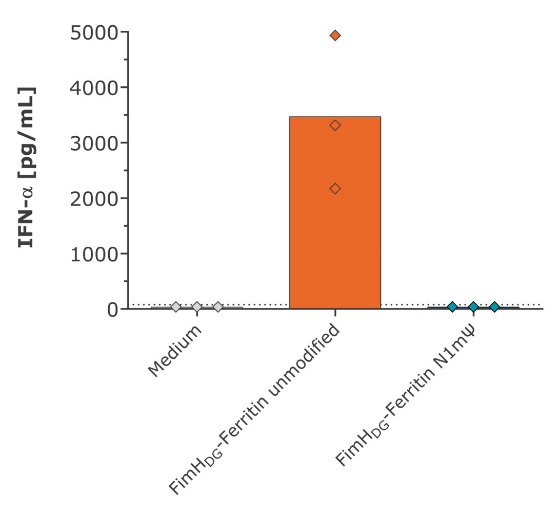


**Supplementary Figure 3. Induction of IFN-α in hPBMCs stimulated with FimH_DG_-Ferritin mRNA vaccines containing unmodified or N1mΨ-modified nucleosides**

hPBMCs from three donors were incubated each in triplicates with 10 µg/ml of LNP-formulated FimH_DG_-Ferritin mRNA vaccines containing either unmodified or modified (N1mΨ) nucleosides. IFN-α levels were detected via an ELISA in cell-free supernatants, harvested 24 h post stimulation. The means derived from the mean values of each triplicate of the three individual donors are depicted. Dotted lines indicate the lower limit of quantification (LLOQ). Values below the LLOQ (78 pg/mL) were set to 39 pg/mL.
